# Induced neural stem cells ameliorate blood-brain barrier injury by modulating the calcium signaling pathway of astrocyte in cerebral ischemia-reperfusion rats

**DOI:** 10.3389/fcell.2025.1611226

**Published:** 2025-06-03

**Authors:** Xueyun Liang, Chuanshang Cao, Ningmei Liu, Dongmei Chen, Ting Liu, Haibin Ma, Jiaxin Liu, Taojuan Wu, Jianguo Niu

**Affiliations:** ^1^ Key Laboratory of Ningxia Stem Cell and Regenerative Medicine, Institute of Medical Sciences, General Hospital of Ningxia Medical University, Yinchuan, China; ^2^ Ningxia Key Laboratory of Cerebrocranial Diseases, School of Basic Medical Sciences, Ningxia Medical University, Yinchuan, China

**Keywords:** induced neural stem cells, BBB, astrocytes, calcium signaling pathways, cerebral ischemia-reperfusion

## Abstract

**Background:**

Neural stem cells offer new hope for ischemic stroke patients on the basis of their potential to reverse neurological sequelae, but it is still difficult to obtain sufficient neural stem cells in the clinic. We induced human placental mesenchymal stem cells to neural stem cells (iNSCs), the therapeutic effects and possible mechanisms of iNSCs in ischemic stroke were observed in this study.

**Results:**

Transplanted iNSCs improved neurological deficits, increased the integrity of blood-brain barrier (BBB) structure and its related proteins expression level in middle cerebral artery occlusion/reperfusion (MCAO/R) rats. The *in vitro* study demonstrated that iNSCs treatment inhibited Ca^2+^ influx in oxygen-glucose deprived (OGD)-damaged astrocytes. Additionally, iNSCs downregulated the expression level of pCaMK-II, increased the expression level of superoxide dismutase, and inhibited the expression of caspase 9 in both brain of MCAO/R rats and OGD-damaged astrocytes.

**Conclusion:**

iNSCs transplantation improved BBB function by modulating calcium signaling pathway of astrocyte in MCAO/R rats, which proved iNSCs may be a new promising neural stem cells origin for the treatment of cerebral ischemia-reperfusion injury.

## 1 Introduction

Cerebral ischemic stroke accounts for most stroke cases, and can lead to death or permanent neurological deficits in severe cases (Collaborators, 2021). Thrombolysis with tissue plasminogen activator and mechanical thrombectomy are the two broad modalities currently available for the treatment of ischemic stroke (Hurd et al., 2021). The key problem with both of these treatments is that they only improve cerebral perfusion and cannot inhibit the additional damage caused by other pathological processes during reperfusion (Paul and Candelario-Jalil, 2021). The interplay of a complex set of pathological processes, including mitochondrial dysfunction, oxidative stress, intracellular calcium overload and excitotoxicity, activation of apoptosis, and inflammation caused by the interruption of cerebral blood flow and subsequent reperfusion, is the main cause of ischemia-reperfusion injury (Awasthi et al., 2024; Zhang et al., 2024). There is an urgent need to explore effective therapeutic methods for treating ischemic stroke during reperfusion (Kuriakose and Xiao, 2020; Ma et al., 2025).

The blood-brain barrier (BBB) is a protective natural barrier between the brain parenchyma and the peripheral circulatory system, and astrocytes are present around the cerebral microvasculature and maintain BBB function through astrocyte-derived factors and the astrocytic end-foot (Ballabh et al., 2004; Wosik et al., 2007). The changing state of astrocytes around injured vessels in ischemic injury disrupts astrocyte-endothelial cell interactions, leading to morphological changes in the BBB and dysfunction (Fattakhov et al., 2024). Therefore, facilitating the repair of the BBB by protecting astrocytes, thereby promoting BBB repair may become an important strategy for the treatment of cerebral ischemia-reperfusion (Takarada-Iemata et al., 2018).

With the development of stem cell research, stem cell therapies, especially neural stem cells (NSCs), have offered new hope for ischemic stroke patients on the basis of their potential to reverse neurological sequelae (Genet and Hirschi, 2021; Buchlak et al., 2024). However, it is still difficult to obtain sufficient NSCs in the clinic. Fortunately, in our previous study, we induced human placental mesenchymal stem cells (PMSCs) into neural stem cells and proved the induced neural stem cells (iNSCs) have potent therapeutic effects on intracerebral hemorrhage in rats (Liu J. et al., 2023).

Our previous study found that iNSCs highly express a variety of neurotrophic factors, such as BDNF, NGF, and GDNF (Liu J. et al., 2023), which were proved to be benefit to the function of astrocytes (Tiberi et al., 2023; Harvey and Rios, 2024; Li et al., 2025). So we hypothesized that iNSCs transplantation could be rescue the BBB function by protection to the function of astrocytes after ischemic stroke. To date, the role of iNSCs in the treatment of ischemic stroke and the associated mechanisms remain unclear. In this study, the therapeutic effects of iNSCs on ischemic stroke were observed in iNSCs-transplanted middle cerebral artery occlusion/reperfusion (MCAO/R) model rats. The possible mechanisms, by which iNSCs maintain their ability to protect the BBB after cerebral ischemia/reperfusion, were also investigated. This study would provide a theoretical support for iNSCs clinical use in future.

## 2 Materials and methods

### 2.1 Ethics and animals

The animals’ ethical protocols and experimental procedures were approved by the Animal Welfare Ethics Review Committee of General Hospital of Ningxia Medical University (No. 2020-725). The adult male Sprague-Dawley rats (250–280 g) used in the experiments were purchased from the Experimental Animal Centre of Ningxia Medical University. The rats were randomly divided into the Sham group (n = 20), MCAO/R group (n = 20), PMSC group (n = 20), and iNSCs group (n = 20). The rats received a standard diet and were given adequate food and water. All the animal experiments were performed in accordance with the Guide lines for the Care and Use of Laboratory Animals.

### 2.2 PMSCs culture

The human placental mesenchymal stem cells (PMSCs) used in the experiments were derived from P3 generation cells provided by the Key Laboratory of Ningxia Stem Cells and Regenerative Medicine of the General Hospital of Ningxia Medical University, and the collection and acquisition of the cells were approved by the Ethics Committee of the General Hospital of Ningxia Medical University (No. 2020-289). PMSCs were cultured with serum-free medium (ultra culture serum-free medium + Pall Ultroser G serum substitute) at 37°C in a 5% CO_2_ incubator.

### 2.3 iNSCs culture

PMSCs of the P3 generation were employed to induce iNSCs. The iNSCs induction medium was configured with the following contents: neurobasal medium-A (97%, Gibco, United States), B27 supplement (2%, Gibco, United States), N-2 supplement (1%, Gibco, United States), bFGF (10 mg/L, Pepro Tech, United States), EGF (20 mg/L, Pepro Tech, United States), and heparin (2 mg/L, Stem Cell, Canada). When the cultured PMSC density reached 80%–90%, the cells were digested with TrypLE™ (1x, Gibco, United States), and 2 × 10^6^ PMSCs were cultured with iNSCs induction medium in a 100 mm culture dish at 37°C in a 5% CO_2_ incubator. The formation of neurospheres was observed via Olympus (Olympus, Japan) phase-contrast microscopy, and 4 mL of fresh iNSCs induction medium was used to replenish the culture dish every 3 days.

### 2.4 Middle cerebral artery occlusion and reperfusion (MCAO/R) model establishment and iNSCs transplantation

The adult male Sprague-Dawley rats were anesthetized with mask inhalation of 3% isoflurane for induction and 1.5% isoflurane for maintenance. Body temperature was maintained at 37°C via a thermostatically controlled heating pad. The right common carotid artery, internal carotid artery (ICA), and external carotid artery (ECA) were exposed. The right ECA was subsequently isolated and ligated. A nylon monofilament with a silicone-coated tip was inserted into the ICA through the external carotid stump and advanced to occlude the middle cerebral artery for 90 min, after which the suture was gently withdrawn. The incision was sutured and the rats were transferred to the cage after awakening. Cerebral blood flow in rats was measured via laser Doppler blood flow assessment throughout the entire procedure of the MCAO/R model establishment.

**FIGURE 1:**
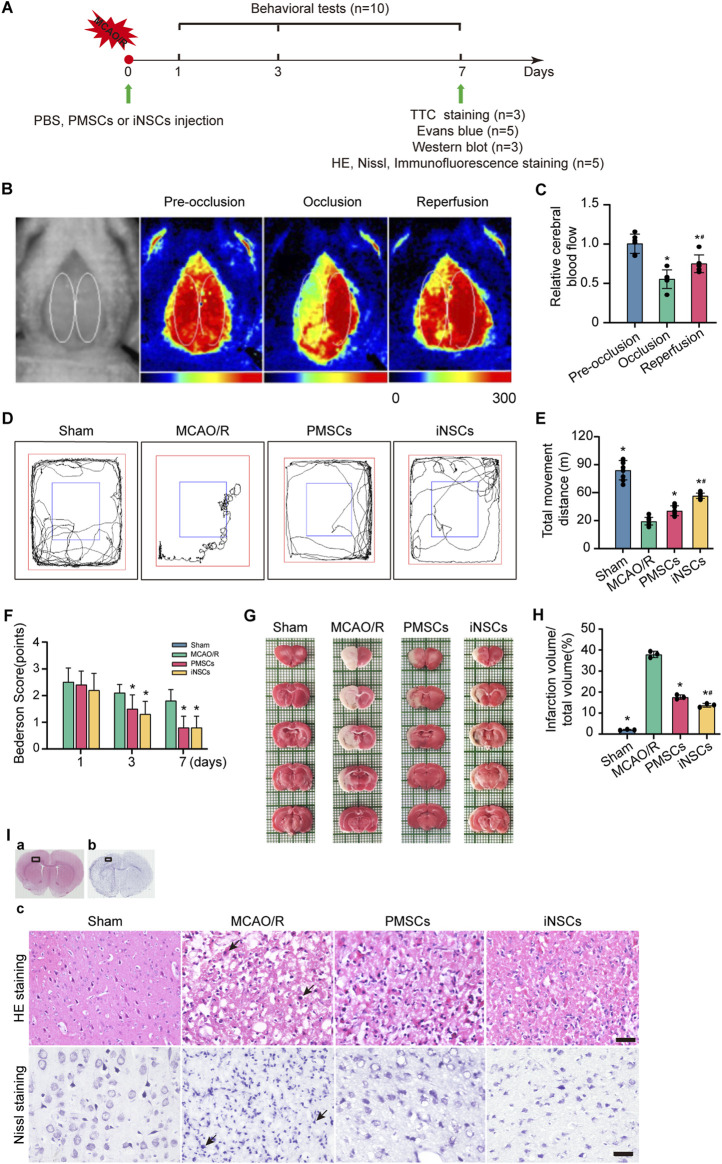
Effects of iNSCs transplantation on MCAO/R rats. **(A)** Schematic drawing about the design of the animal experiments. **(B)** Representative laser scatter images of cerebral blood flow during the entire procedure of the MCAO/R model establishment. **(C)** Quantitative analysis of the cerebral blood flow level during the procedure of the MCAO/R model establishment. The data are expressed as the means ± SDs, (n = 6). **(D)** Representative images of the open field test. **(E)** Quantitative analysis of the movement distance of the open field test in each group. The data are expressed as the means ± SDs, (n = 10). **(F)** Quantitative analysis of the Bederson score test; behavioral evaluation was performed on the 1st, 3rd, and 7th days after cells transplantation in each group. The data are expressed as the means ± SDs, (n = 10). **(G)** Representative images of TTC staining in each group on day 7 after cells transplantation. **(H)** Quantitative analysis of the infarction volume of brain on the 7th days after cells’ transplantation in each group. The data are expressed as the means ± SDs, (n = 3). **(I)** Representative images of HE staining and Nissl staining in each group after cells transplantation. Scale bar = 20 μm (n = 5). Comparisons of means among multiple groups were performed via one-way ANOVA followed by Tukey’s *post hoc* test, and *P < 0.05, compared with the MCAO/R group; ^#^ *P* < 0.05, compared with the PMSCs group.

For the iNSCs or PMSCs group, after the MCAO/R model was established, 1 × 10^6^/30 μL single iNSCs or PMSCs was one-time injected positionally. The stereotaxic coordinates for iNSCs or PMSCs injection were as follows: anteroposterior (AP) 0.24 mm, mediolateral (ML) 4.0 mm, and dorsoventral (DV) 3.5 mm (left). When the injection finished, the needle was maintained for 10 min, and then withdrawn slowly. The needle hole was closed with bone wax, and the wound was sutured. For the MCAO/R groups, the rats were injected positionally with 30 μL of PBS, and the other procedures were similar to those used for the iNSCs or PMSCs group. For the Sham groups, the rats were subjected to the same procedure except that the advancement distance of the monofilament was reduced to 5 mm from the common carotid bifurcation. All the animals were housed and sacrificed individually after 7 days for further analysis (Figure 1A).

### 2.5 Neurobehavioral assessment

On day 7 after cells transplantation, the Open-field test was performed to evaluate the rats’ motor function. Briefly, the rats were gently placed into a corner of the open field experiment box. After the rats had adapted for 1 min, their behavior was observed and recorded for 5 min, and real-time video recording was performed at the same time. The total distance moved during the 5 min was used as a parameter for locomotor performance.

On days 1, 3 and 7 after cells transplantation, the neurological status was evaluated via a five-point scale based on the Bederson score (Liu et al., 2012). The scores were assigned as follows: 0, no observable deficits; 1, forelimb flexion; 2, forelimb flexion and decreased resistance to lateral push; 3, circling; 4, circling and spinning around the cranial-caudal axis; 5, no spontaneous movement.

### 2.6 Cerebral infarction volume assessment

On day 7 after cells transplantation, 2,3,5-triphenyltetrazolium chloride monohydrate (TTC) staining was used to evaluate the infarct volume as previously reported (Bederson et al., 1986). The coronal slices (2 mm thick) of fresh rat brains were immersed in a 2% solution of TTC (Solarbio, China) at 37°C for 10 min in the dark. At the 5th minute, the slices were flipped over. Finally, the slices were soaked in 4% PFA phosphate buffer for 24 h and then digitally photographed. The infarct areas were quantified via ImageJ software. Both direct (Cao et al., 2024) and indirect (Liu T. et al., 2023) measurement were used to calculate the infarct volume, the indirect method is well corrected the brain edema, especially in the first 3 days after ischemia (Lin et al., 1993). Considering the brain edema is not remarkable on day 7 after cells transplantation, we choose the direct method to overestimate the infarct volume as the following formula: infarction volume (%) = (infarct tissue volume/total tissue volume) × 100%.

### 2.7 Evans blue permeability assay

On day 7 after cells transplantation, blood-brain barrier (BBB) disruption was quantitatively determined by Evans blue staining (Gong et al., 2021a). Briefly, 2% Evans blue dye solution (4 mL/kg) was injected through the tail vein 2 h prior to euthanasia. The rats were subsequently anaesthetized with 100 mg/kg pentobarbital intraperitoneal injection and perfused with PBS solution. The brains were rapidly isolated, imaged, weighed and homogenized in formamide (l ml/100 mg tissue). After centrifugation, the supernatant was collected and the absorbance of Evans blue at 620 nm was measured via an enzyme-labeled instrument (Multiskan GO, Thermo, United States). The concentrations of Evans blue in the brain tissue were determined via a standard curve method.

### 2.8 Fixation and sectioning of brain tissue

On day 7 after cells transplantation, the rats were euthanized under deep anesthesia with 100 mg/kg pentobarbital intraperitoneal injection for the purpose of conducting histological and immunofluorescence studies. In essence, the circulatory system of the rats was cleared with PBS via the left ventricle, followed by perfusion with a 4% paraformaldehyde (PFA) solution. The brain tissues were subsequently extracted and immersed in 4% PFA at 4°C for 24 h and then transferred to 30% sucrose solution at 4°C until they sank to the bottom. Thereafter, the brain tissue was embedded in optimal cutting temperature compound and cut into 10 µm-thick coronal sections via a freezing microtome.

### 2.9 HE staining and Nissl staining

HE staining was conducted according to the protocol of the HE Staining Kit (G1120, Solarbio). Briefly, the sections were stained with hematoxylin for 1 min and then soaked in acidic liquid alcohol for 30 s. After being stained with eosin for 50 s and dehydrated with ethanol (95%, 100%), the sections were finally cleared with xylene and mounted. Nissl staining was conducted according to the protocol of Nissl staining kit (G1430, Solarbio). The tissue sections were immersed in tar violet staining solution and stained at 56°C for 1 h. The samples were then washed with deionized water for 5 min and differentiated with Nissl differentiation solution for 2 min until the background turned colorless. Finally, the tissue sections were dehydrated and sealed with neutral resin for preservation. Images were obtained with a microscope (Olympus, Japan).

### 2.10 Immunofluorescence staining

The samples from each group were washed three times with PBS, treated with 0.5% Triton X-100 for 30 min, and then blocked with normal serum for 1 h at room temperature. The sections were subsequently incubated with the following primary antibodies overnight at 4°C: anti-GFAP (1:300, ab7260, Abcam), anti-CaMK-Ⅱ (1:200, ab134041, Abcam), anti-pCaMK-Ⅱ (1:500, ab171095, Abcam), anti- Aquaporin-4 (1:200, ab259318, Abcam), anti-Dystrophin (1:500, ab1764, Abcam), and anti-Syntrophin (1:500, 11425, Abcam). The following day, the sections were incubated with the fluorescence-labeled secondary antibodies Alexa Fluor 555 or Alexa Fluor 488 for 2 h at room temperature in the dark. DAPI staining solution was added to the sections, which were then observed under a fluorescence microscope (Olympus, Japan), and images were acquired. For analysis of the staining results, five samples from each group were selected, three sections were selected from each sample, five areas around the ischemic penumbra were randomly photographed in each section, and the number of positive cells was counted.

### 2.11 Primary astrocyte culture

Primary astrocytes were extracted from neonatal rat brains and cultured as previously described (Liu J. et al., 2023). Briefly, the cerebral cortex tissue was removed from newborn Sprague-Dawley rats and incubated in TrypLE™ at 37°C for 15 min. Cell suspension was prepared by repeated pipetting of digested cortical tissue through different bore sized Pasteur pipettes and strained through 100 μM size cell strainers. Then the cortical cells from three rats were plated on a T-25 flask with Dulbecco’s modified Eagle’s medium (DMEM) supplemented with 10% fetal bovine serum (FBS) and incubated at 37°C with 5% CO_2_ in an incubator. The medium was replaced with fresh medium every 3 days. The purity of the primary astrocytes was determined by glial fibrillary acidic protein (GFAP) immunofluorescence staining.

### 2.12 *In vitro* astrocyte oxygen-glucose deprived (OGD) model establishment

The experiment was divided into 4 groups. Normal group: The astrocytes were cultured with complete medium (DMEM+10% FBS) under normoxia conditions. OGD group: The astrocytes (2 × 10^5^/well) were cultured with glucose-free Dulbecco’s modified Eagle’s medium, and the cells were incubated in a modular hypoxia chamber (1% O_2_, 5% CO_2_, balanced with N_2_) at 37°C for 24 h, then recovered to normoxia conditions with complete medium. PMSC group: The culture condition was the same with that in the OGD group, and the astrocytes were co-cultured with PMSCs for 24 h after OGD. iNSCs group: The culture condition was the same as that in the OGD group, and the astrocytes were cocultured with iNSCs for 24 h after OGD. The samples from the Normal, OGD, PMSCs and iNSCs groups were collected for calcium imaging, immunofluorescence staining, and Western blot analysis (Figure 6A).

### 2.13 Calcium imaging

The cells from each group were washed three times with HBSS after the supernatant was discarded, incubated in 4 μM Fluo-4 working solution at 37°C for 1 h, washed three times with HBSS buffer and incubated in HBSS buffer for another 30 min at room temperature. The images were taken and analyzed every second via fluorescence microscopy (THERMO, United States), and 30% H_2_O_2_ was added after 60 s of baseline measurement. The fluorescence intensities were measured every second via ImageJ and the data were plotted over time.

### 2.14 Western blot analysis

The tissue or cells were manually homogenized, and protein lysates were extracted via IP lysis buffer (Thermo), which included a protease and phosphatase inhibitor cocktail (Thermo). The protein samples were run under reducing conditions. Twenty micrograms of protein lysate were loaded onto 10% or 12.5% SDS‒PAGE gels to separate various types of proteins, wet transferred onto PVDF membranes, blocked with 5% nonfat milk for 2 h and incubated overnight at 4°C with the following primary antibodies: anti-GLUT-1 (1:1000, ab115730, Abcam), anti-Claudin-5 (1:2000, ab131259, Abcam), anti-CaMK-Ⅱ(1:2000, ab134041, Abcam), anti-pCaMK-Ⅱ (1:5000, ab171095, Wako), anti-Aquaporin-4 (1:800, ab259318, Abcam), anti-S100β(1:1000, ab52642, Abcam), anti-Caspase8 (1:1000, ab227430, Abcam), anti-Caspase9 (1:1000, WL01551, Wanlelbio), anti-SOD (1:1000, ab183881, Abcam), anti-β-actin (1:1000, 20536-1-AP, Proteintech), and anti-GAPDH (1:10000, 10494-1-AP, Proteintech). The following day, the membranes were washed three times with TBS-T buffer (10 mM Tris, 150 mM NaCl, 0.05% Tween-20, pH 7.5) for 10 min each time, and then incubated with horseradish peroxidase (HRP)-labeled goat anti-rabbit (1:10000, ab6721, abcam) or goat anti-mouse IgG (1:10000, G1214, Servicebio) secondary antibody, respectively. The following day, the membranes were washed three times with TBS-T buffer (10 mM Tris, 150 mM NaCl, 0.05% Tween-20, pH 7.5) for 10 min each time and then incubated with horseradish peroxidase (HRP)-labeled goat anti-rabbit (1:10000, ab6721, Abcam) or goat anti-mouse IgG (1:10000, G1214, Servicebio) secondary antibodies for 1 h at room temperature. The protein bands on the membranes were developed with an ECL detection kit (Cat. No: KGP1127, KeyGEN BioTECH) and visualized via Amersham Image Quant 800 system (United States). The membranes were stripped for 10 min at RT with Restore PLUS Western blot Stripping Buffer (Thermo Fisher) before being stained for β-actin or GAPDH. Finally, the intensity values of the protein bands from each group were analyzed via ImageJ software and the band intensity values were normalized to the internal control of β-actin or GAPDH and the mean relative changes in protein levels were normalized to those of the control group.

### 2.15 Statistical analysis

All the results were recorded and assessed by a blinded method. Each experiment was repeated at least three times. The Shapiro–Wilk test was used for normally distributed test, and no relevant deviations from normality were found. Comparisons of means among multiple groups were performed via one-way ANOVA followed by Tukey’s *post hoc* test. The data are presented as the means ± SDs. Graphing and statistical analysis were performed via Graph Pad Prism 9.0, and P < 0.05 was considered as statistically significant.

## 3 Results

### 3.1 The MCAO/R model was established

Laser scatter imaging was used to analyze the changes in cerebral blood flow in the rats throughout the entire procedure used to establish the MCAO/R model. The cerebral blood flow level of the rats was significantly decreased at 0.51 ± 0.11 (n = 6, p < 0.05) in the mid-occlusion stage compared to that in the stage of pre-occlusion (0.93 ± 0.11, n = 6). And the cerebral blood flow level of the rats was significantly increased at 0.69 ± 0.10 (n = 6, p < 0.05) in the reperfusion stage compared to that in the mid-occlusion stage (Figures 1B,C).

### 3.2 iNSCs transplantation improved the neurological function in MCAO/R rats

The methods of the open field test and Bederson score were used to investigate the effects of transplanted iNSCs on behavioral functions in MCAO/R rats. The results demonstrated a significant longer movement distance of the open field test in both the PMSCs group and iNSCs group compared to that in the MCAO/R group on day 7 (n = 10, p < 0.05) (Figures 1D,E). The Bederson scores were significantly lower in both the PMSCs group and iNSCs groups than that in the MCAO/R group on day 3 and 7 (n = 10, p < 0.05) (Figure 1F).

### 3.3 iNSCs transplantation reduced the cerebral infarct volume in MCAO/R rats

On the day 7 after cells transplantation, the infarction volume of the rats’ brain in each group was determined via TTC staining. The results showed that the infarction volume of brain was significantly smaller in both the PMSCs group and iNSCs group than that in the MCAO/R group (n = 3, p < 0.05) (Figures 1G,H).

### 3.4 iNSCs transplantation ameliorated neural cell damage in MCAO/R rats

HE and Nissl staining were performed to examine the impact of transplanted iNSCs on the morphological changes in the rats’ brain in each group. HE staining revealed that the arrangement of the cells was disordered, the cytoplasm was condensed, the nuclei were lysed, and many inflammatory cells infiltrated in the infarcted area of the brain in the MCAO/R group. However, compared with the MCAO/R group, the PMSCs and iNSCs groups presented relatively uniform cellular arrangement, reduced cell swelling, and decreased inflammatory cell infiltration in the infarcted area of the brain (Figure 1I).

Additionally, Nissl staining showed that the neuronal cells were arranged in a disordered manner, and most of the cells demonstrated obvious abnormalities, many apoptotic degenerated neural cells with shrunken or fragmented nuclei were observed in the infarcted area of the brain in the MCAO/R group. Compared with those in the MCAO/R group, the PMSCs and iNSCs groups presented relatively regular cell morphology and fewer apoptotic degenerated neural cells in the infarcted area of the brain (Figure 1I).

### 3.5 iNSCs transplantation improved BBB permeability in MCAO/R rats

**FIGURE 2:**
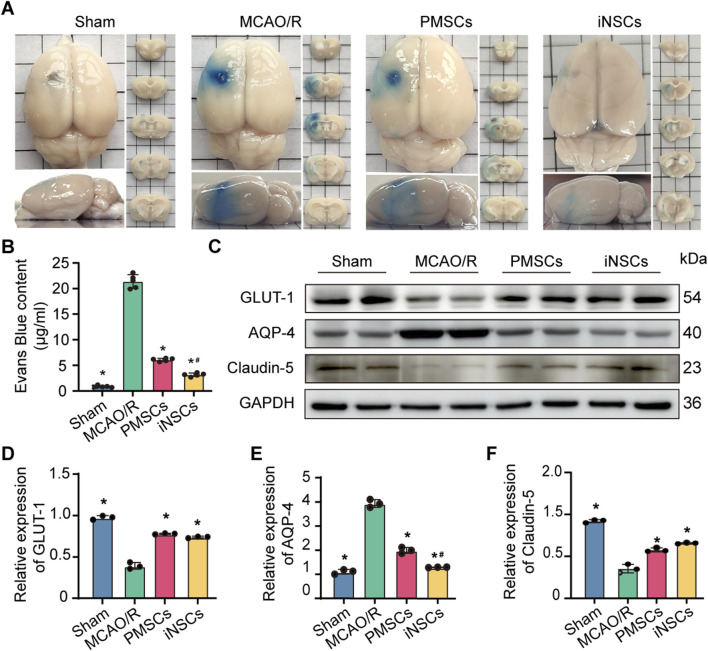
Effects of iNSCs transplantation on BBB function in MCAO/R rats. **(A)** Representative images of Evans blue stained samples from each group. **(B)** Quantitative analysis of the Evans blue content in each group. The data are expressed as the means ± SDs, (n = 5). **(C)** Representative Western blot images of BBB structure-related protein expression in each group. **(D–F)** Results of quantitative analysis of the proteins expression levels of GLUT-1, AQP-4, and Claudin-5, respectively, and were normalized to that of GAPDH. The data are expressed as the means ± SDs, (n = 3). Comparisons of means among multiple groups were performed via one-way ANOVA followed by Tukey’s *post hoc* test, and *P < 0.05, compared with the MCAO/R group; ^#^ *P* < 0.05, compared with the PMSCs group.

The integrity of the BBB was determined by Evans blue staining on day 7 after cells transplantation in each group. The results showed that the content of Evans blue was significantly reduced in the brains of rats from the PMSCs and iNSCs groups compared with that in the MCAO/R group (n = 5, p < 0.05). Furthermore, the content of Evans blue was significantly reduced in the brain from the iNSCs group compared with that in the PMSCs group (n = 5, p < 0.05) (Figures 2A,B).

### 3.6 iNSCs transplantation modulated the expression of BBB structure related proteins in MCAO/R rats

To further observe the effects of iNSCs transplantation on the integrity of BBB, the expression levels of GLUT-1, Aquaporin-4 (AQP4), and Claudin-5, which are related to the structure of the BBB (Kitchen et al., 2020; Zapata-Acevedo et al., 2024), were examined in the brain tissues on day 7 after cells transplantation in each group via the Western blotting. The results revealed that the expression levels of Claudin-5 and GLUT-1 were significantly lower in the MCAO/R groups than in the Sham group, but were greater in the iNSCs group and the PMSCs group than in the MCAO/R group (n = 3, p < 0.05) (Figures 2C,D,F). In contrast, the expression of AQP-4 was greater in the MCAO/R groups than in the Sham group, but it was lower in the iNSCs group and the PMSCs group than in the MCAO/R group (n = 3, p < 0.05) (Figures 2C,E).

### 3.7 iNSCs transplantation affected the distribution of AQP-4 around the BBB in MCAO/R model rats

**FIGURE 3:**
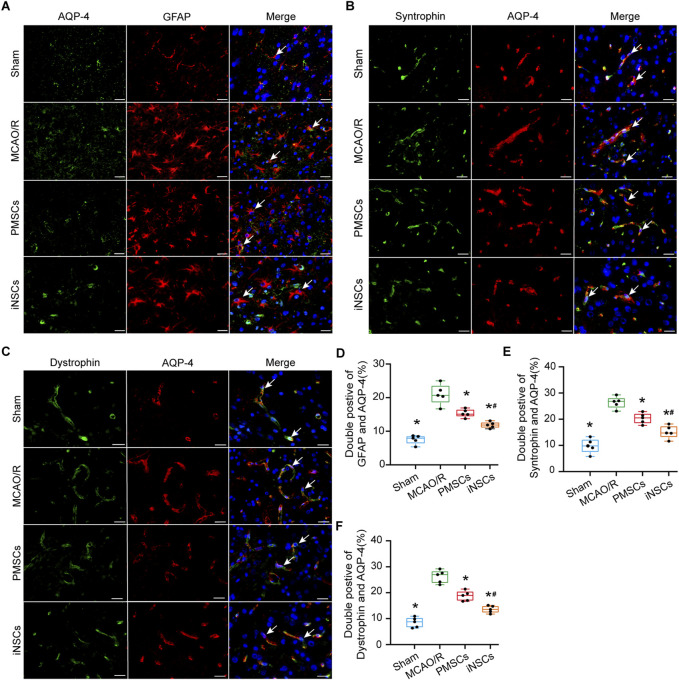
Effects of iNSCs transplantation on AQP4 expressions in MCAO/R rats. **(A–C)** Representative images of immunofluorescence double staining of AQP-4 with either GFAP, Syntrophin or Dystrophin in each group. Scale bar = 20 μm. **(D–F)** The results of quantitative analysis results of double positive cells of AQP-4 with either GFAP, Syntrophin or Dystrophin in each group. The data are expressed as the means ± SDs, (n = 5). Comparisons of means among multiple groups were performed via one-way ANOVA followed by Tukey’s *post hoc* test, and *P < 0.05, compared with the MCAO/R group; ^#^ *P* < 0.05, compared with the PMSCs group.

AQP4 is a water channel protein that links astrocytic endfeet to the BBB and is also recognized as a protein that associated with damage of BBB (Zhu A. et al., 2022). Syntrophin and Dystrophin are known as markers for the polarization of AQP-4, which is localized around the BBB (Neely et al., 2001). Immunofluorescence double staining of AQP-4 with either GFAP, Syntrophin or Dystrophin was performed in the brain tissue of each group on day 7 after cells transplantation to observe the effects of iNSCs on the function of AQP-4. Compared with those in the Sham group, the number of AQP-4 and GFAP double positive cells in the infarcted area of the brain in the MCAO/R groups was significantly greater (n = 5, p < 0.05), and the number of AQP-4 and GFAP double positive cells in the infarcted area of the brain in the iNSCs and PMSCs groups was significantly lower than that in the MCAO/R group (n = 5, p < 0.05) (Figures 3A,D). Similar results were shown for both the double staining of AQP-4 with Syntrophin and AQP-4 with Dystrophin (n = 5, p < 0.05) (Figures 3B,C,E,F).

### 3.8 iNSCs transplantation affected pCaMK-II/ CaMK-II expression levels in the brain tissue of MCAO/R rats

**FIGURE 4:**
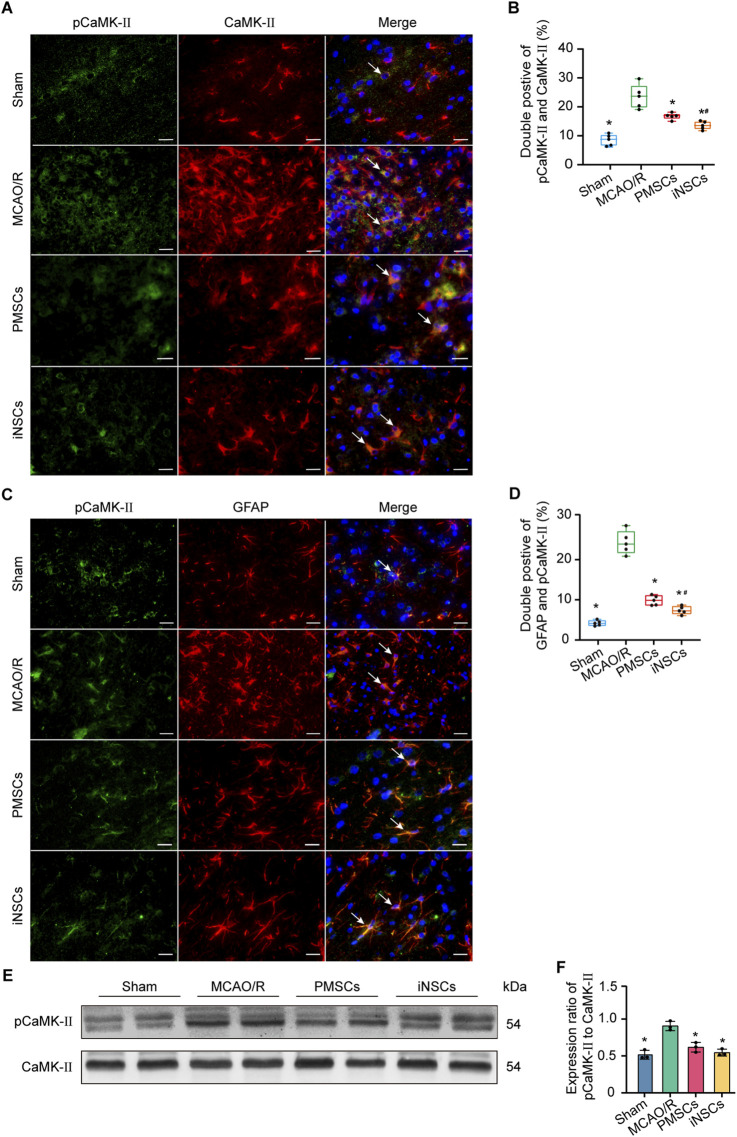
Effects of iNSCs transplantation on pCaMK-II expression in MCAO/R rats. **(A)** Representative images of immunofluorescence double staining of pCaMK-II with CaMK-II in each group. Scale bar = 20 μm. **(B)** Quantitative analysis results of pCaMK-II and CaMK-II double positive cells in each group. The data are expressed as the means ± SDs, (n = 5). **(C)** The representative images of immunofluorescence of pCaMK-II with GFAP double staining in each group. Scale bar = 20 μm. **(D)** Results of Quantitative analysis of the double positive cells of pCaMK-II with GFAP in each group. The data are expressed as the means ± SDs, (n = 5). **(E)** Representative Western blot images of pCaMK-II and CaMK-II expression in each group. **(F)** Results of quantitative analysis of the proteins expression ratio of pCaMK-II to CaMK-II, and normalization to one of the values from the MCAO/R group. The data are expressed as the means ± SDs, (n = 3). Comparisons of means among multiple groups were performed via one-way ANOVA followed by Tukey’s *post hoc* test, and *P < 0.05, compared with the MCAO/R group; ^#^ *P* < 0.05, compared with the PMSCs group.

The expression levels of phosphorylated calmodulin-dependent protein kinase II (pCaMK-II) and calmodulin-dependent protein kinase II (CaMK-II), which are related to the calcium signaling pathway (Tokumitsu and Sakagami, 2022), were examined in brain tissues on day 7 after cells transplantation in each group via immunofluorescence double staining and Western blotting. The number of pCaMKII and CaMK-II double positive cells in the infarcted area of the brain in the MCAO/R groups was significantly increased compared with that in the Sham group (n = 5, p < 0.05), and the number of pCaMKII and CaMK-II double positive cells in the infarcted area of the brain in the iNSCs and PMSCs groups was significantly decreased compared with that in the MCAO/R group (n = 5, p < 0.05) (Figures 4A,B). Similarly, the Western blot results showed that the expression levels’ ratio of pCaMK-II to CaMK-II were significantly increased in the MCAO/R groups compared with that in the Sham group, but it was decreased in the iNSCs group and the PMSCs group compared with that in the MCAO/R group (n = 3, p < 0.05) (Figures 4E,F).

### 3.9 iNSCs transplantation affected the expression of pCaMK II in astrocytes

Double immunofluorescence staining of pCaMKII and GFAP was conducted in the brain tissue of each group on day 7 after cells transplantation to investigate the effect of iNSCs transplantation on the expression of calcium signaling pathway-related proteins in astrocytes. The results showed that the number of pCaMKII and GFAP double positive cells in the infarcted area of the brain in the MCAO/R groups was significantly increased compared with that in the Sham group (n = 5, p < 0.05), and the number of pCaMKII and GFAP double positive cells in the infarcted area of the brain in the iNSCs and PMSCs groups was significantly decreased compared with that in the MCAO/R group (n = 5, p < 0.05) (Figures 4C,D).

### 3.10 iNSCs transplantation protected cellular mitochondrial function in MCAO/R model rats

**FIGURE 5:**
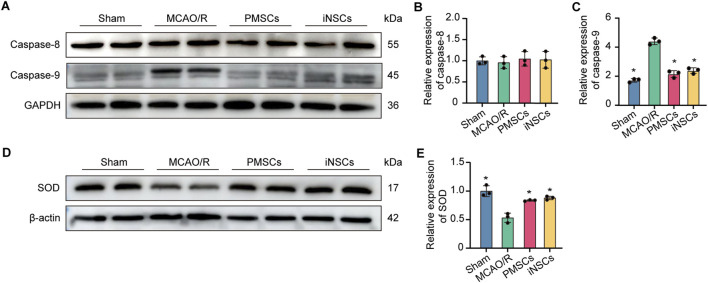
Effects of iNSCs transplantation on mitochondrial function in MCAO/R rats. **(A)** Representative Western blot images of Caspase 8 and Caspase 9 expression in each group. **(B,C)** Quantitative analysis of the expression level of Caspase 8 and Caspase 9, respectively, and normalized to GAPDH. **(D)** Representative Western blot images of SOD expression in each group. **(E)** Quantitative analysis of the expression level of SOD, which was normalized to β-actin. The data are expressed as the means ± SDs, (n = 3). Comparisons of means among multiple groups were performed via one-way ANOVA followed by Tukey’s *post hoc* test, and *P < 0.05, compared with the MCAO/R group; ^#^ *P* < 0.05, compared with the PMSCs group.

The expression levels of Caspase 8, Caspase 9 and SOD were measured in the brain tissues on day 7 after cells transplantation in each group by the Western blotting to analyze the effect of iNSCs transplantation on the cellular mitochondrial function. The results showed that the expression levels of Caspase 9 were significantly increased in the MCAO/R groups compared with that in the Sham group, but it was decreased in the iNSCs group and the PMSCs group compared with that in the MCAO/R group (n = 3, p < 0.05) (Figures 5A,C). Contrarily, the expression of SOD was decreased in the MCAO/R groups compared with that in the Sham group, but it was increased in the iNSCs group and the PMSCs group compared with that in the MCAO/R group (n = 3, p < 0.05) (Figures 5D,E). Comparatively, there was no difference in the expression level of Caspase 8 among the groups (n = 3) (Figures 5A,B).

### 3.11 iNSCs treatment affected AQP-4 expression in OGD-damaged astrocytes

**FIGURE 6:**
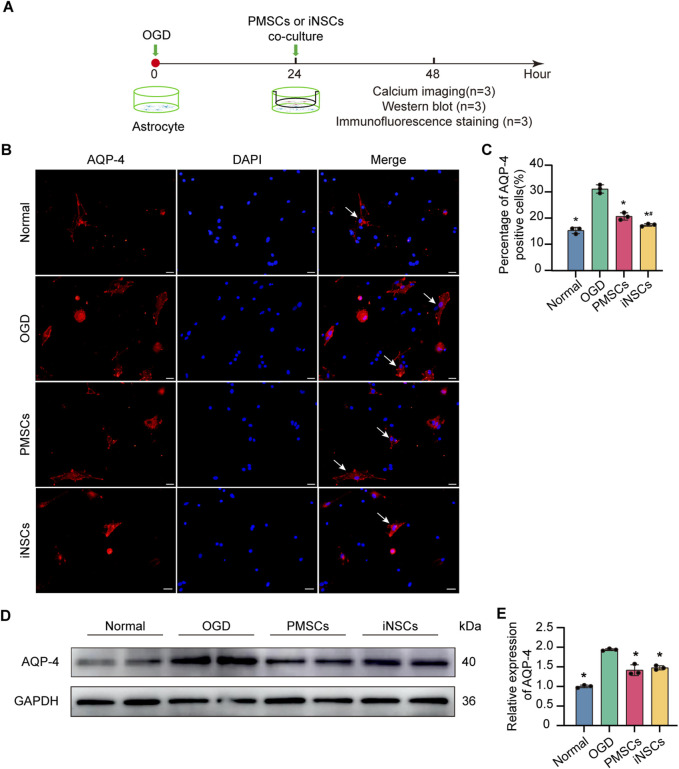
Effects of iNSCs treatment on AQP-4 expression in OGD-damaged astrocytes. **(A)** Schematic drawing of the design of the *in vivo* experiments. **(B)** Representative images of immunofluorescence staining of AQP-4 in astrocytes from each group. Scale bar = 20 μm. **(C)** Quantitative analysis of the percentage of AQP-4 positive astrocytes in each group. The data are expressed as the means ± SDs, (n = 3). **(D)** Representative Western blot images of AQP-4 expression in each group. **(E)** Quantitative analysis of the expression level of AQP-4, normalized to that of GAPDH. The data are expressed as the means ± SDs, (n = 3). Comparisons of means among multiple groups were performed via one-way ANOVA followed by Tukey’s *post hoc* test, and *P < 0.05, compared with the OGD group; ^#^ *P* < 0.05, compared with the PMSCs group.

Immunofluorescence staining of AQP-4 was performed in the astrocytes from each group to observe the distribution of AQP-4 in the astrocytes. The results showed that the percentage of AQP-4 positive cells were significantly increased in the OGD groups compared with that in the Normal group, but it was decreased in the iNSCs group and the PMSCs group compared with that in the OGD group (n = 3, p < 0.05) (Figures 6B,C).

Next, the expression level of AQP-4 in astrocytes in each group was determined via the Western blotting. The results revealed that the expression level of AQP-4 was significantly increased in the OGD groups compared with that in the Normal group, but it was decreased in the iNSCs group and the PMSCs group compared with that in the OGD group (n = 3, p < 0.05) (Figures 6D,E).

### 3.12 iNSCs treatment regulated calcium influx in OGD-damaged astrocytes

**FIGURE 7:**
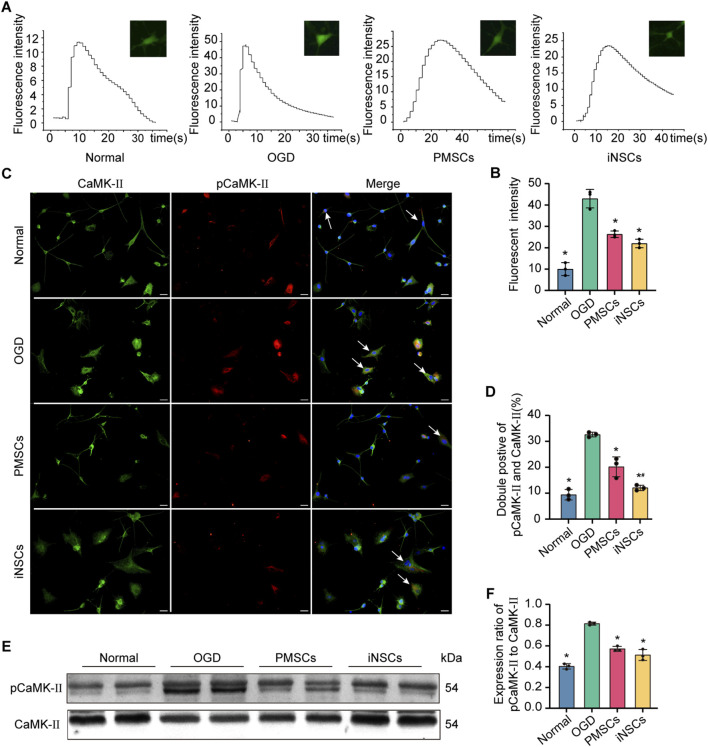
Effects of iNSCs treatment on the calcium signaling pathway in OGD damaged astrocytes. **(A,B)** Representative images of calcium imaging in astrocytes from each group and quantitative analysis of the intracellular calcium in astrocytes from each group. **(C,D)** Representative images of immunofluorescence double staining of pCaMK-II and CaMK-II in astrocytes from each group and quantitative analysis of the percentage of pCaMK-II and CaMK-II positive cells in each group. The arrows indicate co-labeled positive cells, scale bar = 20 μm. **(E)** Representative Western blot images of pCaMK-II and CaMK-II expression in each group. **(F)** Quantitative analysis of the expression level of pCaMK-II, which was normalized to that of CaMK-II. The data are expressed as the means ± SDs, (n = 3). Comparisons of means among multiple groups were performed via one-way ANOVA followed by Tukey’s *post hoc* test, and *P < 0.05, compared with the OGD group; ^#^P < 0.05, compared with the PMSCs group.

Calcium imaging of astrocytes from each group was conducted to observe the intracellular calcium flow induced by H_2_O_2_ stimulation. The results revealed that the peak of fluorescence intensity, which was related to the quantity of intracellular calcium, was significantly decreased in the iNSCs and PMSCs groups compared with that in the OGD group (n = 3, p < 0.05) (Figures 7A,B).

### 3.13 iNSCs treatment affected calcium signaling pathway related protein expression in OGD-damaged astrocytes

The expression levels of pCaMK-II and CaMK-II were examined in astrocytes from each group by the immunofluorescence double staining and Western blotting. The number of pCaMKII and CaMK-II double positive cells in the OGD groups was significantly increased compared with that in the Normal group (n = 3, p < 0.05), and the number of pCaMKII and CaMK-II double positive cells in the iNSCs and PMSCs groups was significantly decreased compared with that in the OGD group (n = 3, p < 0.05) (Figures 7C,D). Similarly, the Western blot results showed that the expression levels’ ratio of pCaMK-II to CaMK-II were significantly increased in the OGD groups compared with that in the Normal group, but it was decreased in the iNSCs group and the PMSCs group compared with that in the OGD group (n = 3, p < 0.05) (Figures 7E,F).

### 3.14 iNSCs treatment protected mitochondrial function in OGD-damaged astrocytes

**FIGURE 8:**
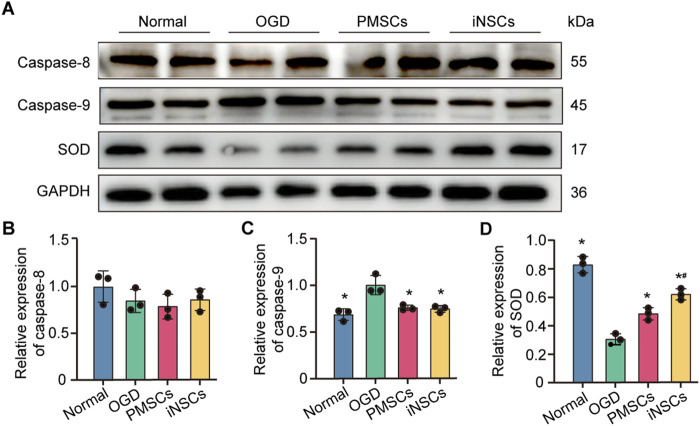
Effects of iNSCs treatment on mitochondrial function in OGD damaged astrocytes. **(A)** Representative Western blot images of Caspase 8, Caspase 9 and SOD expression in each group. **(B–D)** Quantitative analysis of the expression levesl of Caspase 8, Caspase 9 and SOD, respectively, and normalization to the level of GAPDH. The data are expressed as the means ± SDs, (n = 3). Comparison of means among multiple groups was performed using one-way ANOVA followed by Tukey’s *post hoc* test, and *P < 0.05, compared with the OGD group; ^#^ *P* < 0.05, compared with the PMSCs group.

The expression levels of Caspase 8, Caspase 9 and SOD were measured in astrocytes from each group by the Western blotting to analyze the effect of iNSCs treatment on the mitochondrial function in OGD-damaged astrocytes. The results showed that the expression levels of Caspase 9 were significantly increased in the OGD groups compared with that in the Normal group, but it was decreased in the iNSCs group and the PMSCs group compared with that in the OGD group (n = 3, p < 0.05) (Figures 8A,C). In contrast, the expression of SOD was decreased in the OGD groups compared with that in the Normal group, but it was increased in the iNSCs group and the PMSCs group compared with that in the OGD group (n = 3, p < 0.05) (Figures 8A,D). Comparatively, there was no difference of the expression level of Caspase 8 among the groups (n = 3) (Figures 8A,B).

## 4 Discussions

Ischemic stroke is the leading cause of disability and death, but the drugs currently used to treat ischemic stroke are insufficient to effectively improve patient recovery (Kim et al., 2020; Malik et al., 2020). Multiple studies have shown that NSCs present a promising treatment as a multifaceted neuroprotective strategy to ameliorate neural injury (Tang et al., 2023; Li et al., 2024), but it is difficult to obtain enough human NSCs for clinical use. Our previous study proved that PMSCs can be induced into iNSCs, and it highly express NSC-specific markers, such as SOX-2 and Nestin. Meanwhile, it could be differentiated into neuron- and glia-like cells *in vitro* and *in vivo* (Liu J. et al., 2023). In this study, our results showed that transplanted iNSCs could improve neurological deficits, such as recover of motor function, cerebral area infarction, and neural cells damage. Our study confirmed the previous findings that NSC transplantation could effectively promote structural repair and functional recovery in MCAO/R models (Wang et al., 2024). These results supported that iNSCs might be a new origin of NSC, which could be used for ischemic stroke treatment in the future.

BBB damage is an important pathological feature of ischemic stroke and can further exacerbate brain damage (Gong et al., 2021b). When an ischemic stroke results in the functional impairment of cellular components, the disruption of tight junction proteins, such as Claudin-5 and GLUT-1, could lead to an increase in the permeability of the BBB (Yang et al., 2021). Our study showed that iNSCs transplantation reduced the permeability of Evans blue and increased the expression levels of the BBB structure-related proteins, caudin-5 and GLUT-1, which indicated that iNSCs have a restorative effect on BBB damage after cerebral ischemia-reperfusion.

Astrocytes are among the major cells that make up the BBB and play a role in regulating the integrity of the BBB. Aquaporin 4 (AQP-4) is widely distributed on the end-foot of astrocytes and is involved in BBB development and integrity, its increased expression and polarization aggravate cerebral edema, which in turn exacerbates BBB damage (Li et al., 2013; Zhu D. D. et al., 2022). Loss of AQP-4 polarization is a hallmark of a wide range of brain pathologies associated with stroke (Frydenlund et al., 2006). We found that iNSCs treatment reduced the expression of AQP-4 in astrocytes in both MCAO/R rat brain and OGD damaged culture. Moreover, iNSCs transplantation can reduce the expression of AQP-4 around the BBB, which in turn reduces the extent of damage to the BBB structure. Taken together, these results suggest that iNSCs can repair BBB injury after cerebral ischemia/reperfusion, possibly through intervention targeting AQP-4.

A growing body of evidence has demonstrated that the distribution of AQP-4 polarity is related to astrocytic calcium signaling (Kitchen et al., 2020). Our results demonstrated that iNSCs treatment inhibited Ca^2+^ influx in OGD-damaged astrocytes, thus alleviating the damage caused by intracellular Ca^2+^ accumulation in OGD-damaged astrocytes. It has been shown that intracellular Ca^2+^ can activate the calmodulin-dependent protein kinase II (CaMK-II) pathway, leading to apoptotic cell death (Takano et al., 2003; Park et al., 2022). Activated CaMK-II maintains its active form throughout the process of autophosphorylation (Swulius and Waxham, 2008). Lee et al. reported that ROS-mediated Ca^2+^ levels are involved in the activation of CaMKII (Lee et al., 2021). We further proved *in vivo* and *ex vivo* that iNSCs downregulated the expression level of pCaMK-II in astrocytes both in MCAO/R rats and OGD damaged culture. These results suggest that iNSCs might play a role in regulating the intracellular calcium signaling pathway.

Calcium signaling can manipulate ROS levels, which can directly or indirectly affect mitochondrial functions (Giorgi et al., 2018). After ischemic stroke, excessive accumulation of ROS further aggravates ischemia-reperfusion injury by activating mitochondria-mediated apoptosis and promoting the degradation of tight junction proteins, which disrupts the integrity of the BBB (He et al., 2020). Superoxide dismutase (SOD), which is related to mitochondrial function, is a major antioxidant in the body that reduces the excessive accumulation of reactive oxygen species and thus repairs the oxidative damage caused by free radicals (Niizuma et al., 2010). SOD inhibits hypoxia-induced vasodilation and blood-brain barrier damage and attenuates endothelial cell damage (Tu et al., 2014). Caspase 9 has been shown to be a marker of mitochondrial damage (Shalini et al., 2015). Our results showed that iNSCs transplantation increased the expression level of SOD both in MCAO/R rats and in OGD damaged astrocytes. Moreover, iNSCs transplantation inhibited caspase 9 expression in MCAO/R rats and in OGD damaged astrocytes. All of the above results suggest that iNSCs transplantation could improve mitochondrial function in astrocytes, possibly due to the role of iNSCs’ in calcium signaling modulation.

On the basis of our results, we speculated that iNSCs transplantation improved the BBB function after cerebral ischemia/reperfusion possibly by modulating the astrocyte calcium signaling pathway. Thus, iNSCs may be a promising tool for the treatment of cerebral ischemia-reperfusion injury in the future.

The limitations of this study are that the current results can only prove the correlation between iNSCs and the calcium pathway, but the iNSCs suppression target on the calcium pathway was not clarified, the further experiments still needed to verify the detail mechanism.

## Data Availability

The datasets presented in this study can be found in online repositories. The names of the repository/repositories and accession number(s) can be found in the article/supplementary material.

## Abbreviations

AQP-4, Aquaporin-4; BBB, Blood-brain barrier; CaMK-II, Calmodulin-dependent protein kinase II; ECA, External carotid artery; FBS, Fetal bovine serum; GFAP, Glial fibrillary acidic protein; iNSCs, Induced neural stem cells; ICA, Internal carotid artery; MCAO/R, Middle cerebral artery occlusion/reperfusion; OGD, Oxygen-glucose deprived; pCaMK-II Phosphorylated calmodulin-dependent protein kinase II; PFA, Paraformaldehyde; PMSCs, Placental mesenchymal stem cells; SOD, Superoxide dismutase; TTC, 2,3,5-triphenyltetrazolium chloride monohydrate.
